# Association between serum elastin-derived peptides and abdominal aortic calcification in peritoneal dialysis patients: a cross-sectional study

**DOI:** 10.1080/0886022X.2021.1918163

**Published:** 2021-05-17

**Authors:** Shizhu Zhao, Jingyuan Cao, Jianzhong Li, Xiaochun Yang, Peiyang Cao, Jingjing Lan, Guoyuan Lu

**Affiliations:** a Department of Nephrology, The First Affiliated Hospital of Soochow University, Suzhou, China; b Department of Nephrology, Taizhou People’s hospital, The Fifth Affiliated Hospital of Nantong University, Taizhou, China; c Department of Radiology, The Second Affiliated Hospital of Soochow University, Suzhou, China; d Department of Nephology, Wuxi Traditional Chinese Medicine Hospital, Wuxi, China

**Keywords:** Peritoneal dialysis, abdominal aortic calcification, elastin-derived peptides, elastin

## Abstract

**Background:**

Peritoneal dialysis (PD) patients experience accelerated arterial aging, which is characterized by elastin degradation. Elastin-derived peptides (EDPs) are direct products of elastin fragmentation. This study tried to explore the association between serum EDPs and abdominal aortic calcification (AAC) in PD patients.

**Methods:**

Serum levels of EDPs were analyzed in 126 eligible PD patients and 30 controls. PD patients were grouped according to the annularity of AAC evaluated by an abdominal computed tomography (CT) scan. Serum EDPs were analyzed in relation to the presence of AAC or severe AAC in PD patients by logistic regression analysis.

**Results:**

Serum EDPs in PD patients were significantly higher than age-matched controls. In 126 PD patients, higher EDPs was associated with greater risk of present AAC (OR = 1.056, 95%CI 1.010–1.103) and severe AAC (OR = 1.062, 95%CI 1.004–1.123). A combination of EDPs substantially improved the accuracy of diagnostic performance for AAC and severe AAC.

**Conclusions:**

EDPs can predict the presence and extent of AAC in PD patients, indicating its possible role to recognize PD patients at risk for AAC and severe AAC.

## Introduction

Cardiovascular disease (CVD) remains one of the major causes of death in patients starting peritoneal dialysis (PD) [1], and those with vascular calcification are thought to have the highest risk of future CVD [2]. The prevalence of abdominal aortic calcification (AAC) among dialysis patients in Asia is up to 46.8 − 85.71% [3–5]. Potential biomarkers to improve AAC risk stratification may help understand its pathogenesis and even become targets.

Vascular calcification is thought to be a process of ageing accompanied by obvious extra-cellular matrix remodeling featured by elastin degradation [6]. Concurrently with aging, the increased sympathetic activity, local inflammation, and oxidative stress could activate proteases including cysteine proteinases and matrix metalloproteinases (MMPs) [7,8], and irreversibly degrades elastin into elastin-derived peptides (EDPs) [9].

In uremic patients with accelerated arterial aging, elastodystrophy of aorta occurred early and constructed a context of altered mineral metabolism for the pathogenesis of arterial medial calcification [10]. In dialysis patients with more metabolic, hemodynamic, inflammatory, and hormonal changes [11], elastic fiber fragmentation in epigastric artery was more obvious when compared with healthy kidney donors [12]. These findings indicated PD patients underwent accelerated elastin degradation. In aneurysms featured by elastin breakdown, structural products of EDPs and desmosine in plasma increased and correlated with aneurysm rupture and aneurysm-related death [13,14]. Thus, we hypothesized EDPs in PD patients might also be elevated and correlate with AAC. In this study, we measured serum EDPs and evaluated its association with AAC in PD patients detected by a multi-slice spiral CT scan (MSCT).

## Materials and methods

The study included adult PD patients and healthy volunteers from August 2018 to January 2020 in the First Affiliated Hospital of Soochow University. All participants signed an informed consent, and the study was approved by the ethics committee in accordance with the Declaration of Helsinki.

### Study population

**Figure 1.**
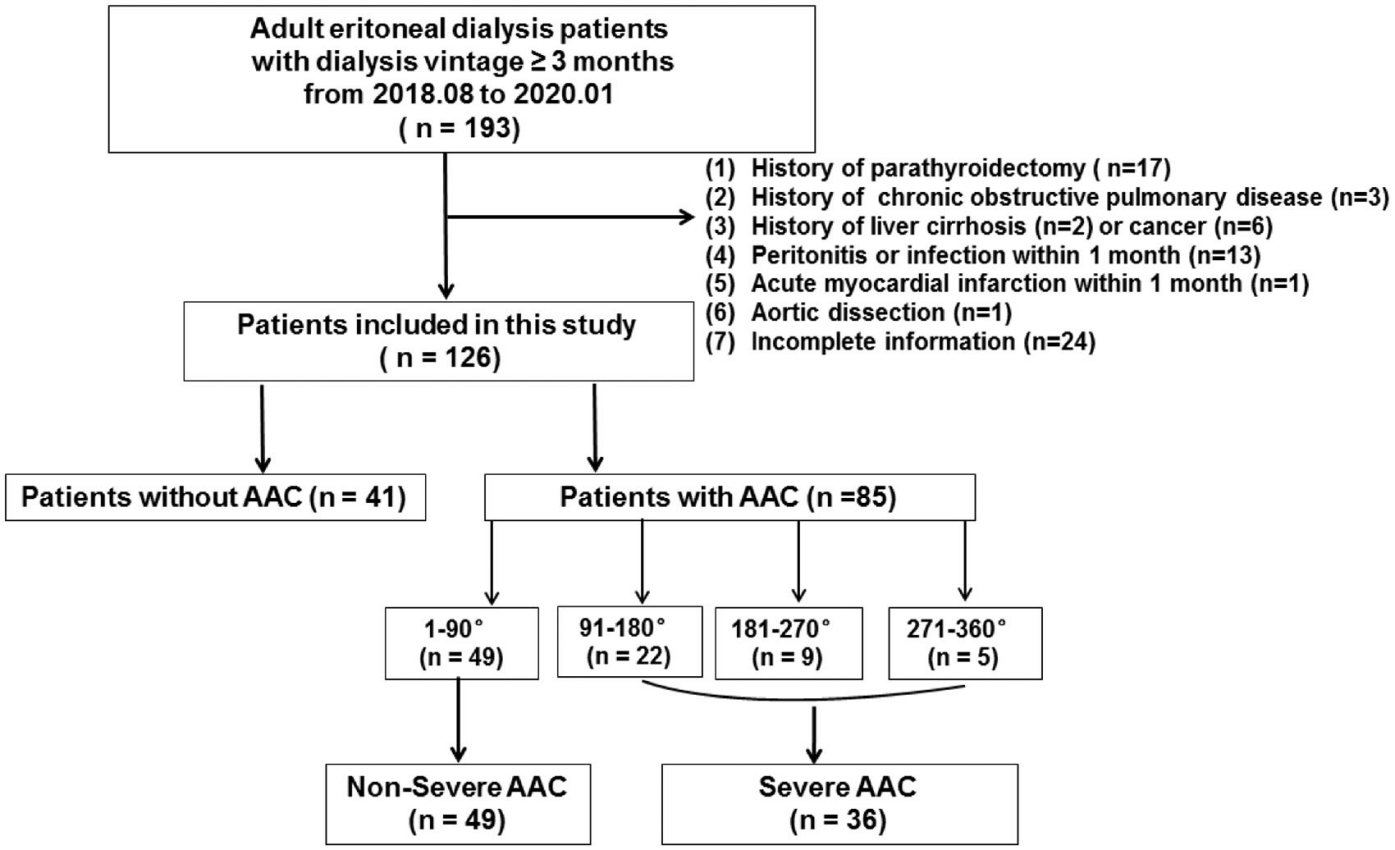
Flowchart of study participants for the cross-sectional study. AAC indicates abdominal aortic calcification.

PD patients receiving continuous ambulatory peritoneal dialysis with low calcium (1.25 mmol/L) peritoneal dialysis solution (Baxter Healthcare CO.LTD, Guangzhou, China) for at least three months were included. Exclusion criteria were (1) history of parathyroidectomy, (2) chronic obstructive pulmonary disease (COPD), (3) liver cirrhosis, (4) cancer, (5) peritonitis or infection within 1 month, (6) acute myocardial infarction or aortic dissection within 1 month, and (7) incomplete information on AAC evaluation. The flowchart was displayed in Figure 1.

At the same time, 30 healthy subjects were randomly selected among age- and sex-matched people undergoing medical examination in the Medical Examination Center. Every of them had a normal glomerular filtration rate and underwent a CT scan to rule out the presence of AAC. They also had no history of proteinuria, hematuria, hypertension, or diabetes.

### Evaluation of AAC

**Figure 2.**
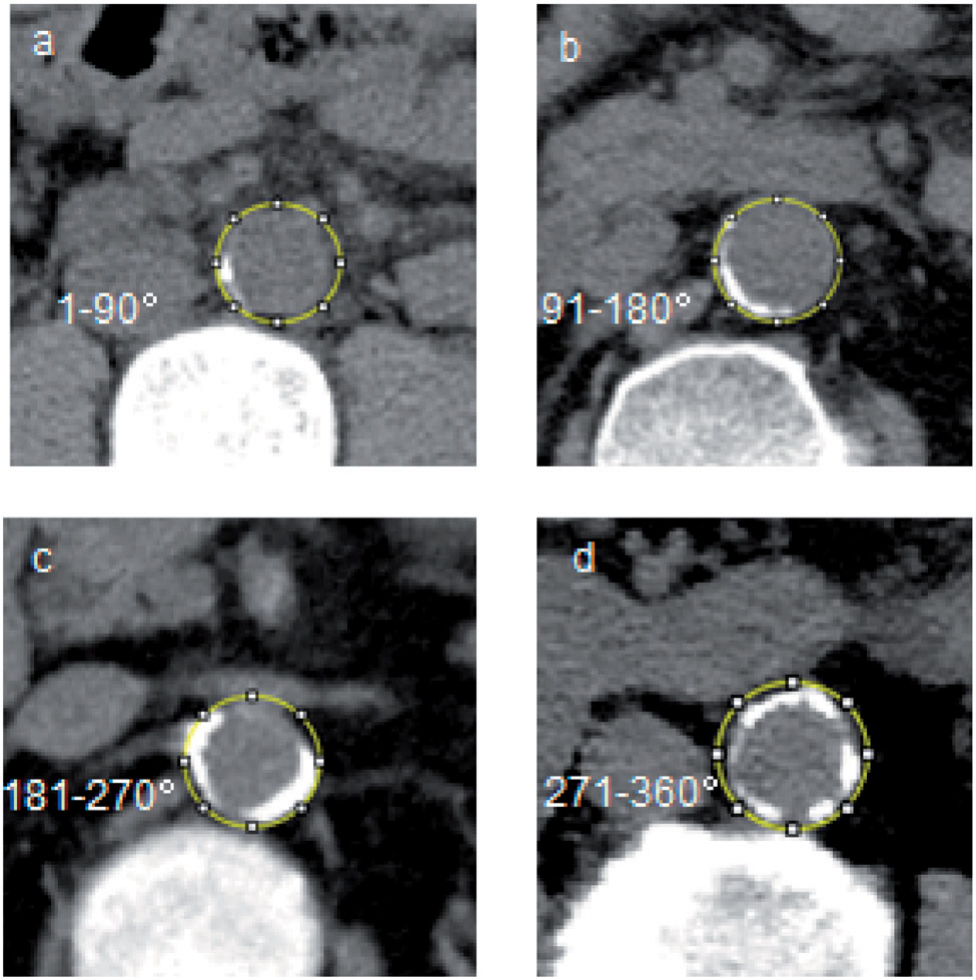
Cross-sectional view of abdominal aortic calcification by CT in four representative cases.

All patients received noncontrast abdominal MSCT during hospitalization, and the detailed CT protocol was presented in Online Resource 1. AAC was defined as calcified plaques with area ≥1mm^3^ and density ≥130 Hounsfield unit [15] along the abdominal aorta walking path from diaphragm to the common iliac artery. Calcification of abdominal aorta was measured on cross-sectional images by one author (Xiao-chun Yang, with 20 years of radiology research experience and blinded to our study). Annularity was scored into five categories as absent, 1–90, 91–180, 181–270, and 271–360 degree, which was a simple method highly correlated with the Agatston score quantification [16]. Examples of the semi-quantitative patterns were shown in Figure 2.

### Blood sampling and analysis

Venous samples were collected for at least 10-h fast. After clotting for 30 min at room temperature, the samples were centrifuged at 4000 *g* for 15 min at 4 °C. The upper serum was separated and stored at −80 °C. Until the investigation, serum samples were thawed at room temperature. Specific competitive enzyme-linked immunosorbent assay kits (Aviscera bioscience, USA) were used to measure serum EDPs. The principle and assay procedures were summarized in Online Resource 1.

### Data collection

Age, gender, body mass index (BMI), dialysis vintage (PD vintage), weekly urea clearance (total Kt/V), and blood pressure were recorded on purpose. At the same time, baseline comorbid conditions of hypertension, type-2 diabetes mellitus (T2DM), and CVD consisting of coronary artery disease, cerebral artery disease, and aortic aneurysm were abstracted from previous medical records, as well as the related medications.

Hemoglobin (HB), neutrophil cells (NE), platelets (PLT), lipid profile, creatinine (Cr), β2-microglobulin (β2-MG), uric acid (UA), albumin (Alb), and high sensitivity-C reactive protein (hs-CRP), mineral metabolism parameters including calcium (Ca), phosphate, intact parathyroid hormone (iPTH) and alkaline phosphatase (ALP) [17] were obtained with routine laboratory methods at the beginning of study. Serum albumin in our hospital was measured by Bromcresol green method, and when albumin was lower than 34 g/L, serum calcium was corrected with the formula: corrected Ca = uncorrected Ca + (34 – [Alb (g/L)]) * 0.0176 [18,19].

### Statistical analysis

The continuous variables were expressed as mean ± standard deviation (SD) or median (Q1 – Q3). One-way analysis of variance (ANOVA) or Kruskal–Wallis H test, and independent sample T-test or Mann–Whitney test were used appropriately. Categorical variables were presented as frequencies (percentages) and compared by the Chi-square test or Fisher’s exact test when necessary. Spearman correlation analysis was used to find association of EDPs with other parameters.

PD patients were firstly assigned into AAC versus non-AAC group, and a logistic regression analysis was conducted among PD patients to examine the relationship between EDPs with AAC. We also conducted a subgroup analysis in patients with AAC. Considering the imbalanced sample size, patients were further grouped into severe versus non-severe group with the cutoff annular of 90 degree, as shown in Figure 1. Receiver operation characteristic (ROC) curves were used to determine optimal cutoff values of EDPs by maximizing the Youden index (sensitivity + specificity − 1). Additionally, for sensitivity analysis, EDPs were transferred to a dichotomous variable according to the respective cutoff point of two ROC curves, and then again fully adjusted. A probability of < 0.05 was considered statistically significant for all analyses. All of the analyses were conducted with the IBM SPSS (version 22.0, IBM Inc., NY, USA).

## Results

### Participants

Finally, our study enrolled 126 patients on PD and 30 healthy controls. Patients and controls were comparable in age and sex ratio; however, serum EDPs in PD patients were significantly higher than that in controls. Hyperlipidemia was absent in PD patients, while disorders of mineral parameters were more prevalent (higher serum P and ALP). Detailed demographic and clinical characteristics of all PD patients are summarized in Table 1.

**Table 1.** Comparisons of demographic data and laboratory parameters among PD patients with or without AAC and the controls.

	Control	All PD patients	Non-AAC	AAC
	*N* = 30	*N* = 126	*N* = 41	*N* = 85
Age, year	47 ± 8	50 ± 13	41 ± 11	55 ± 11^b, c^
Male *n*,(%)	17 (60.7)	75 (59.5)	23 (56.1)	52 (61.2)
SBP, mmHg	119 ± 9^a^	147 ± 18	145 (139 − 156)^c^	145 (136 − 155)^c^
DBP, mmHg	72 ± 10^a^	85 ± 12	89 ± 14^c^	83 ± 10^b, c^
BMI, kg/m^2^	NA	23.3± 3.2	22.5 (21.3 − 26.2)	23.4 (21.3 − 25.0)
HBP n, (%)	0	125 (99.2)	41 (100.0)	84 (98.8)
T2DM n, (%)	0	12 (9.5)	3 (7.3)	9 (10.6)
CVD n, (%)	0	9 (7.1)	2 (4.9)	7 (8.2)
PD vintage, month	NA	22.5 (7.0 − 51.0)	12.0 (5.5 − 24.5)	39.0 (9.0 − 61.5)^b^
total Kt/V	NA	1.81 (1.62 − 2.05)	1.74 (1.51 − 2.17)	1.86 (1.67 − 2.04)
HB, g/L	135 ± 19^a^	99 ± 16	102 ± 17^c^	98 ± 16^c^
NE, 10^9^/L	3.5(2.9 − 4.4)	3.7(2.8 − 4.6)	3.4 (2.6 − 4.4)	3.7(2.8 − 4.6)
PLT, 10^6^/L	237 ± 64^a^	171 ± 56	183 ± 55^c^	166 ± 56^c^
TG, mmol/L	1.2 (0.9 – 1.6)	1.4 (1.0 – 2.0)	1.4 (1.1 – 1.9)	1.4 (1.0 – 2.0)
TC, mmol/L	4.57 ± 0.93	4.41 ± 1.09	4.44 ± 1.07	4.39 ± 1.10
HDL, mmol/L	1.12 (0.93 − 1.31)^a^	0.97 (0.81 − 1.19)	0.95 (0.79 − 1.21)	0.98 (0.82 − 1.19)
LDL, mmol/L	2.82 ± 0.85^a^	2.20 ± 0.78	2.24 ± 0.69^c^	2.18 ± 0.82^c^
Cr, µmol/L	63 ± 13^a^	944 ± 292	991 ± 303^c^	921 ± 286^c^
β2-MG, mg/L	NA	28.3 (20.820.8 − 34.3)	27.2 ± 11.3	29.1 ± 10.5
UA, mmol/L	315 ± 100^a^	390 ± 72	416 ± 80^c^	379 ± 65^b, c^
Alb, g/L	44.0 (41.3 − 46.0)^a^	34.6 (31.3 − 37.13)	34.9 ± 3.5^c^	33.3 ± 5.1^b, c^
Ca, mmol/L	2.29 ± 0.13	2.25 ± 0.19	2.16 ± 0.14	2.16 ± 0.20
P, mmol/L	1.24 ± 0.27^a^	1.63 ± 0.40	1.71 ± 0.45^c^	1.59 ± 0.37^c^
hs-CRP, mg/L	1.9 (0.8 − 3.3)	1.9 (0.7 − 4.3)	1.7 (0.4 − 4.8)	2.0 (0.9 − 4.0)
ALP, U/L	57 (47 − 70)^a^	80 (63 − 116)	66 (54 − 94)^c^	88 (71 − 136)^b, c^
iPTH, ng/L	NA	397 (26 − 595)	358 (224 − 542)	405 (266 − 656)
EDPs, ng/mL	31.7 (24.2 − 39.2)^a^	46.0 (37.7 − 55.0)	37.7 (32.3 − 46.5)^c^	48.3 (42.8 − 57.4)^b,c^

Data are presented as mean ± SD, median (Q1–Q3), or frequency (percentage). NA means not assessed. ^a^*p* < 0.05 when comparing between two groups of all PD patients and controls. ^b^*p* < 0.05 when comparing between two groups of PD patients with and without AAC. ^c^*p* < 0.05 in the post-hoc analysis between AAC group with controls or non-AAC group with controls.

AAC indicates abdominal aortic calcification; PD: peritoneal dialysis; SBP: systole blood pressure; DBP: diastole blood pressure; BMI: body mass index; T2DM: type 2 diabetes mellitus; CVD: cardiovascular disease; Kt/V: weekly urea clearance; HB: hemoglobin; NE: neutrophil; PLT: platelet; TG: triglyceride; TC: total cholesterol; HDL: high-density lipoprotein; LDL: low-density lipoprotein; Cr: creatinine; β2-MG: β2-microglobulin; UA: uric acid; Alb: albumin; Ca: calcium; P: phosphate; hs-CRP: high sensitivity C reactive protein; ALP: alkaline phosphatase; iPTH: intact parathyroid hormone; EDPs: elastin-derived peptides.

### Comparisons of characteristics among PD patients with or without AAC

**Figure 3.**
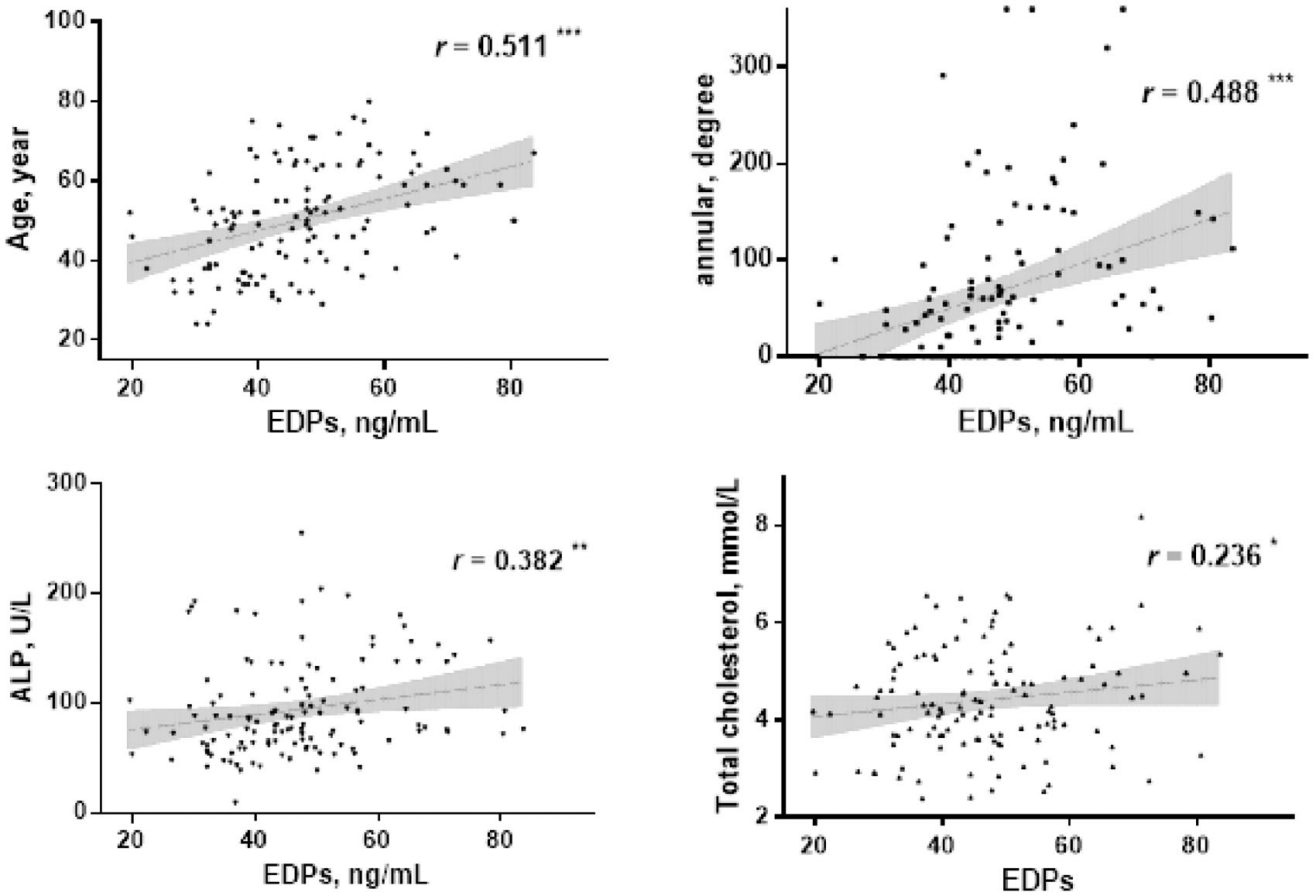
Correlation analyses of EDPs with other parameters in PD patients. ^*^p < 0.05, ^**^p < 0.01, ^***^p < 0.001

The mean age of all PD patients was 50 ± 13 years and the median dialysis vintage was 22.5 (7.0 − 51.0) months. Correlation analysis showed positive association of EDPs with age (*r* = 0.511), annularity of AAC (*r* = 0.488), ALP (*r* = 0.382) and TC (*r* = 0.236) as shown in Figure 3.

**Figure 4.**
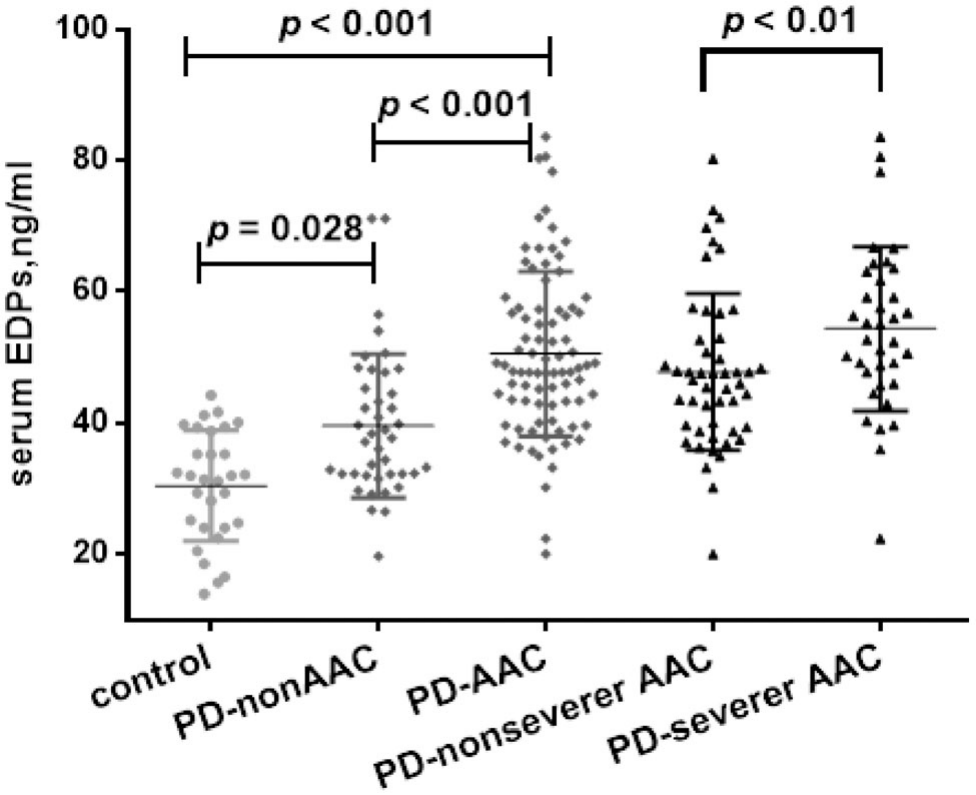
Comparisons of EDPs among different groups presented as a dot-plot. EDPs of PD patients with or without AAC were greatly higher than healthy controls. In all PD patients, EDPs of AAC were significantly higher than those without AAC. In patients with severer AAC, EDPs were also higher than those without severer AAC. AAC indicates abdominal aortic calcification; PD: peritoneal dialysis; EDPs, elastin-derived peptides.

We divided all patients into two groups according to the presence or absence of AAC. 85 (67.5%) patients were in the AAC group, and 41 (32.5%) were within the non-AAC group. As shown in Figure 4, patients with AAC presented higher serum EDPs than those without AAC. The AAC group also exhibited higher age, longer PD vintage and lower DBP, along with higher UA and lower albumin than non-AAC group. Prevalence of T2DM tended to be higher in AAC group, but was not significant. We found no obvious differences in mineral metabolism parameters with the exception for ALP (Table 1).

### Prediction values of EDPs and mineral metabolism parameters for AAC

ROC curves of EDPs and mineral bone disorder parameters showed that EDPs had the most excellent power to discriminate between PD patients with or without AAC. The area under the curve (AUC) of EDPs was 0.767, which was the largest (Online Resource 1, Figure S2).

### EDPs as a predictor of AAC

To examine the association between serum EDPs and AAC in PD patients, eligible covariates with *p* < 0.1 in univariate regression, along with mineral metabolism parameters, total cholesterol (TC), total triglyceride (TG), and history of T2DM were incorporated into the multivariate regression model. After fully adjusted, higher EDPs (OR = 1.056), along with higher age (OR = 1.097), and longer PD vintage (OR = 1.042) were associated with greater odds of AAC in PD patients. However, UA was also a significant determinant (OR = 0.987).

**Figure 5.**
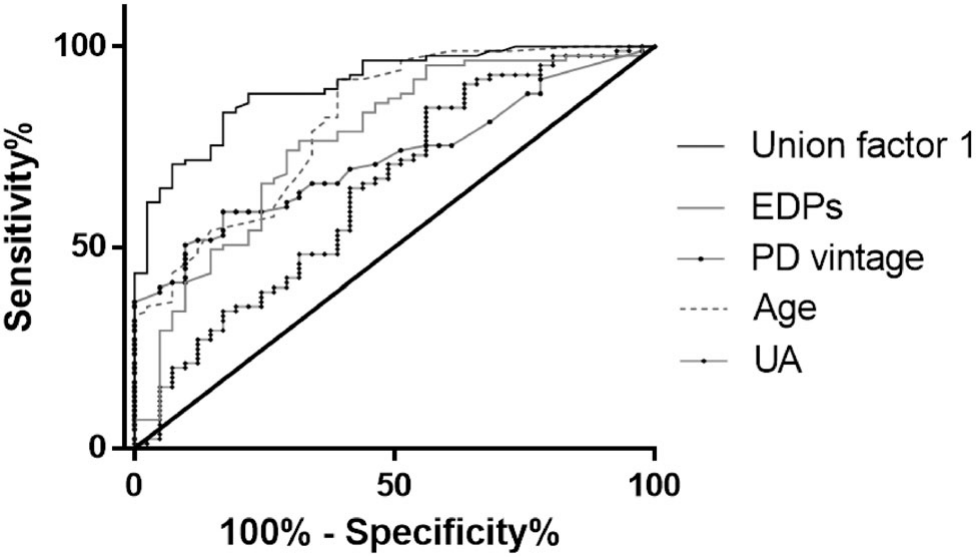
Receiver operating characteristic curves to predict AAC in PD patients. A combined model of age, PD vintage, UA, and EDPs yielded a significant increase in AUC when compared with other factors alone (Union factor 1, AUC = 0.903). The respective AUC of other parameters including EDPs, PD vintage, age, and UA was 0.766, 0.721), 0.813), and 0.645.

ROC curve analysis showed that the cutoff point of EDPs to predict AAC was 43.22 ng/mL, and the sensitivity and specificity values were 74.1% and 70.7%. When EDPs were transferred to a categorical variate according to the cutoff point, OR of EDPs increased to 2.810 (Table 2). A combined model of age, PD vintage, UA, and EDPs yielded a significant increase in AUC when compared with other factors alone (Figure 5).

**Table 2.** Univariate and multivariate logistic regression analysis for effects of EDPs on AAC in PD patients.

	Parameters	Β	SE	Wald	OR	95% CI	*p* Value
Univariate logistic regression	Age, year	0.115	0.023	25.860	1.122	1.073–1.173	<0.001
	DBP, mmHg	−0.043	0.017	6.414	0.958	0.927–0.990	0.011
	PD vintage, month	0.040	0.011	14.311	1.041	1.019–1.062	<0.001
	UA, mmol/L	−0.008	0.003	6.958	0.992	0.986–0.998	0.008
	Alb, g/L	−0.077	0.044	3.039	0.926	0.850–1.010	0.081
	ALP, U/L	0.013	0.013	0.005	6.092	1.003–1.024	0.014
	EDPs, ng/mL	0.086	0.021	16.477	1.090	1.046–1.137	<0.001
Multivariate logistic regression	Age, year	0.093	0.027	11.942	1.097	1.041–1.156	0.001
	PD vintage, month	0.041	0.014	8.368	1.042	1.013–1.071	0.004
	EDPs, ng/mL	0.054	0.022	5.793	1.056	1.010–1.103	0.016
	UA, mmol/L	−0.013	0.004	10.307	0.987	0.979–0.995	0.001
Sensitivity analysis	Age, year	0.099	0.027	13.695	1.104	1.048–1.164	<0.001
	PD vintage, month	0.036	0.013	7.303	1.037	1.010–1.065	0.007
	EDPs (categorical data)	1.015	0.517	3.899	2.760	1.003–7.596	0.049
	UA, mmol/L	−0.012	0.004	8.899	0.988	0.981–0.996	0.003

Binomial logistic regression was analyzed with prevalent AAC as the outcome in all PD patients. The multivariate logistic regression was adjusted for age, DBP, PD vintage, history of T2DM, Alb, UA, Ca, P, ALP, iPTH, TC, and TG. Sensitivity analysis was conducted with EDPs included as a dichotomous variable according to its cutoff point in the ROC curve. OR means per 1 unit increment in an independent variable can lead to the change in the risk of AAC.

AAC indicates abdominal aortic calcification; PD: peritoneal dialysis; DBP: diastole blood pressure; Alb: albumin; Ca: calcium; P: phosphate; ALP: alkaline phosphatase; iPTH: intact parathyroid hormone; TG: triglyceride; TC: total cholesterol; UA: uric acid; EDPs: elastin-derived peptide; OR: odds ratio; and CI: confidence interval.

### Baseline characteristics of PD patients stratified by the severity of AAC

Of all 85 PD patients with AAC, 36 (42.35%) patients were categorized into the severe group with AAC annular > 90 degree, and 49  (57.65%) were in the nonsevere one. When compared with each other, patients with severe AAC were older with longer PD vintage and higher EDPs. Noticeably, classical risk factors of vascular calcification such as phosphate, iPTH and hs-CRP were significantly higher in severe AAC group. They also tended to have higher uremic toxicity biomarkers of β2-MG and Cr (Table 3). At the same time, the use of statin was more frequent among the severe group (Online Resource 1, Table S2).

**Table 3.** Comparisons of demographic data and laboratory parameters with or without severer AAC in PD patients.

	PD		*p* Value
	Non severer AAC	Severer AAC	
	*N* = 49	*N* = 36	
Age, year	51 ± 11	60 ± 10	<0.001^***^
Male *n*,(%)	30 (61.2)	22 (61.1)	1.000
SBP, mmHg	142 (135 − 148)	148 (142 − 160)	0.010^*^
DBP, mmHg	81 (75 − 87)	86 (79 − 89)	0.068
BMI, kg/m2	22.9 ± 2.8	23.3 ± 2.7	0.456
Hypertension *n*,(%)	49 (100.0)	35 (97.2)	0.424
T2DM *n*,(%)	5 (10.2)	4 (11.1)	1.000
CVD *n*,(%)	3 (6.1)	4 (11.1)	0.45
PD vintage, month	12.0 (6.0 − 39.0)	58.5 (43.0 − 79.5)	<0.001^***^
total Kt/V	1.98 (1.64 − 2.26)	1.81 (1.67 − 1.99)	0.118
HB, g/L	97 ± 14	97 ± 16	0.886
NE, 109/L	3.6 (2.8 − 4.6)	3.7 (2.9 − 5.1)	0.584
PLT, 106/L	163 ± 59	169 ± 52	0.666
TG, mmol/L	1.8 ± 1.2	1.5 ± 1.0	0.297
TC, mmol/L	4.39 ± 1.04	4.40 ± 1.20	0.996
HDL, mmol/L	1.02 ± 0.30	1.04 ± 0.32	0.692
LDL, mmol/L	2.19 ± 0.82	2.17 ± 0.81	0.920
Cr, µmol/L	863 ± 299	1000 ± 251	0.029^*^
β2-MG, mg/L	26.4 ± 11.1	32.7 ± 8.6	0.004^**^
UA, mmol/L	369 ± 57	391 ± 75	0.134
Alb, g/L	34.2 ± 5.2	32.2 ± 4.9	0.084
Ca, mmol/L	2.14 ± 0.19	2.18 ± 0.23	0.494
P, mmol/L	1.52 ± 0.38	1.68 ± 0.34	0.049^*^
hs-CRP, mg/L	1.7 ( 0.6 − 3.1)	2.5 (1.9 − 4.7)	0.018^*^
ALP, U/L	80 (69 − 138)	91 (74 − 127)	0.434
iPTH, ng/L	352 (245 − 535)	479 (361 − 714)	0.018^*^
EDPs, ng/mL	47.6 (39.2 − 53.9)	53.8 (46.4 − 62.8)	0.003^**^

Data are presented as mean ± SD, median (interquartile ranges), or frequency (percentage). ^*^p < 0.05,^ **^p < 0.01, ^***^p < 0.001. AAC indicates abdominal aortic calcification; PD, peritoneal dialysis; SBP, systole blood pressure; DBP, diastole blood pressure; BMI, body mass index; T2DM, type-2 diabetes mellitus; CVD, cardiovascular disease; Kt/V, weekly urea clearance; HB, hemoglobin; NE, neutrophil; PLT, platelet; TG, triglyceride; TC, total cholesterol; HDL, high-density lipoprotein; LDL, low-density lipoprotein; Cr, creatinine; β2-MG, β2-microglobulin; UA, uric acid; Alb, albumin; Ca, calcium; P, phosphate; hs-CRP, high sensitivity C reactive protein; ALP, alkaline phosphatase; iPTH, intact parathyroid hormone; EDPs, elastin-derived peptide.

### EDPs as a predictor of severe AAC

**Figure 6.**
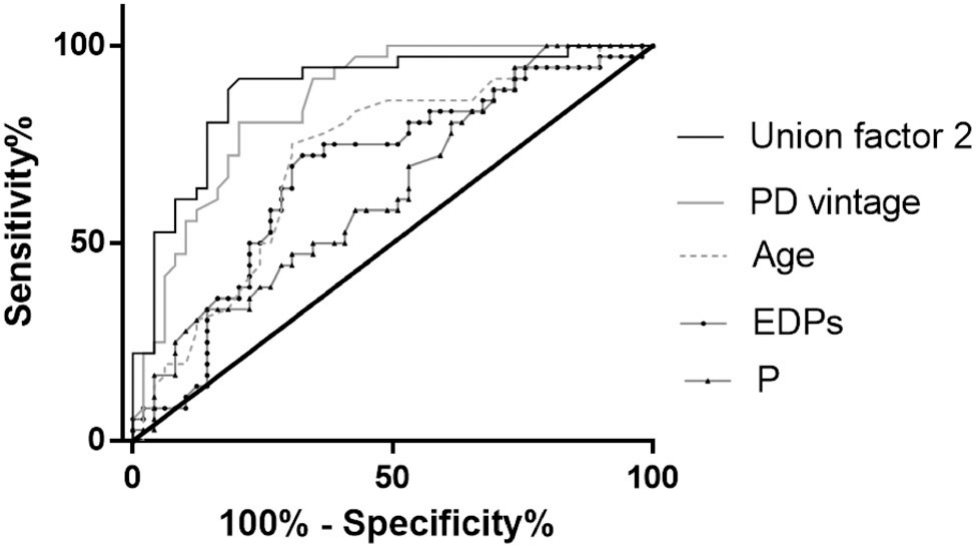
Receiver operating characteristic curves to predict severe AAC in PD patients. A combined model of age, PD vintage, P, and EDPs yielded a significant increase in AUC when compared with other factors alone (Union factor 2, AUC = 0.886). The respective AUC of other parameters including EDPs, PD vintage, age, and P was 0.681, 0.856, 0.715, and 0.633.

As shown in Table 4, after fully adjusted, higher EDPs (OR = 1.062) was also associated with greater odds of severe AAC. The cutoff point of EDPs to predict severe AAC was 48.55 ng/mL, with a sensitivity of 72.2% and a specificity of 67.3%. When EDPs was again used in a binary fashion in the model, its association with severe AAC remained significant (OR = 6.521, Table 4). After incorporation of age, PD vintage, phosphate, and EDPs, the AUC increased to 0.886 to predict severe AAC (Figure 6).

**Table 4.** Logistic regression analysis of EDPs on severer AAC in PD patients.

	parameters	β	SE	Wald	OR	95% CI	*p* Value
Univariate logistic regression	Age, year	0.069	0.022	9.697	1.072	1.026–1.120	0.002
	PD vintage, month	0.047	0.011	19.146	1.048	1.026–1.070	<0.001
	Total Kt/V	−1.029	0.553	3.469	0.357	0.121–1.055	0.063
	Cr, µmol/L	0.002	0.001	5.405	1.002	1.000–1.004	0.010
	β2-MG, mg/L	0.071	0.024	8.750	1.074	1.024–1.126	0.003
	P, mmol/L	1.348	0.649	4.315	3.851	1.079–13.740	0.038
	iPTH, ng/L	0.017	0.008	5.546	1.017	1.001–1.033	0.033
	EDPs, ng/mL	0.045	0.019	5.397	1.046	1.007–1.086	0.020
Multivariate logistic regression	Age, year	0.101	0.034	8.719	1.106	1.034–1.182	0.003
	PD vintage, month	0.043	0.012	13.418	1.044	1.020–1.069	<0.001
	P, mmol/L	3.237	1.192	7.379	25.468	2.464–263.288	0.007
	EDPs, ng/mL	0.060	0.029	4.394	1.062	1.004–1.123	0.036
Sensitivity analysis	Age, year	0.093	0.035	7.087	1.097	1.025–1.175	0.008
	PD vintage, month	0.044	0.012	12.683	1.044	1.020–1.070	<0.001
	P, mmol/L	3.088	1.203	6.585	21.931	2.074–231.928	0.010
	EDPs, ng/mL	1.875	0.692	7.337	6.521	1.679–25.322	0.007

Binomial logistic regression was analyzed with prevalent severer AAC as the outcome in patients with AAC. The multivariate logistic regression was adjusted for age, SBP, PD vintage, total kt/V, T2DM, Cr, β2-MG, P, and iPTH. Sensitivity analysis was conducted with EDPs included as a dichotomous variable according to the cutoff point in its ROC curve to predict severer AAC. OR means per 1 unit increment in an independent variable can lead to the change in the risk of severe AAC.

AAC indicates abdominal aortic calcification; PD: peritoneal dialysis; SBP: systole blood pressure; T2DM: type 2 diabetic mellitus; Cr: creatinine; β2-MG: β2-microglobulin; P: phosphate; iPTH: intact parathyroid hormone; EDPs: elastin-derived peptide; OR: odds ratio; and CI: confidence interval.

## Discussion

The evaluation of cardiovascular calcification has been the focus in dialysis patients as it predicts a higher risk of CVD [2]. PD patients with severe baseline AAC showed greater risk of cardiovascular events and death during follow-up compared with no or mild calcification [20–22], which strengthened the importance of AAC risk stratification. In our study, AAC detected by nonenhanced MSCT is prevalent in 67.5% PD patients, higher than that evaluated by lateral lumber radiography [5]. Most importantly, we highlighted the role of EDPs as a potential biomarker of AAC risk assessment in PD patients. EDPs had the greatest discriminatory power to differentiate the presence of AAC compared with mineral metabolism parameters. Its elevation was associated with higher risk of AAC and severe AAC in PD patients.

Elastin degrades irreparably with aging, and circulating concentration of EDPs increased steadily throughout the human life span [23]. Patients with chronic kidney disease (CKD) experience early vascular ageing [24], and higher EDPs in PD patients than in the age-matched controls were found in our study.

Elastin degradation participates in the pathogenesis of atherosclerosis and medial calcification [25,26], both of which coexist in CKD. EDPs in CKD patients were significantly higher than in controls and were associated with increased aortic stiffness and all-cause mortality [27]. Desmosine was also found to correlate with coronary artery calcification rather than emphysema in patients with COPD [28], indicating EDPs was more relevant to elastin of the vascular rather than the lungs. Apart from that, circulating EDPs were also considerably elevated in diabetes with microvascular complications such as albuminuria and retinopathy [29]. Similar to those researches of strong correlation between EDPs and vascular disease, we found serum EDPs was significantly higher in PD patients with AAC, and it increased as the calcificaiton worsened. Further, elevated EDPs was a vital factor related to the risk of AAC and severe AAC in PD patients, prompting its important role in AAC risk stratification.

The strongest correlation of age with increased EDPs in PD patients of our study also suggested that elastin degradation might also be age-related, but further accelerated in those patients [30–32]. Activation of elastases, such as MMPs and cathepsin, were observed in the abdominal aorta of uremic mice, along with obvious elastin loss [30,31]. While inhibition of the enzymes alleviated elastin loss and might attenuate vascular calcification [31,32]. Further activation of elastases might be a critical component in linking dialysis and the elevation of EDPs. On the other hand, immature elastogenesis might also play a role in the elevation [33,34]. Healthy arteries contain little tropoelastin, the dominant building block of elastic fibers. However, specific labeled molecular MRI targeting tropoelastin suggested a significant accumulation of tropoelastin in atherosclerosis progression [33], which failed to transfer to mature elastin fibers [6] and could be nonspecifically catalyzed by elastases [34], leading to further accumulation of EDPs. However, the abnormal elastogenesis in PD patients needs further research.

Vascular calcification is such a complex process that other factors participating it should also be taken when evaluating the risk of AAC. We found that longer dialysis vintage played an important role in both AAC and severe AAC of PD patients. This was identical to several larger studies, in which duration of dialysis was related to premature AAC evaluated by lateral lumber radiography or conduit arteries medial calcification detected by ultrasound [35–37]. Besides, we detected uric acid as a protective factor in AAC. Consistently, Lai *et al* also found higher SUA was associated with lower cardiovascular and all-cause mortality in 492 PD patients [38]. While a U-shaped relationship between UA and all-cause mortality was found in a nationwide cohort study in Japan [39]. The role of UA in vascular calcification in PD patients seemed to be controversial and needs further investigation.

At the same time, there exist several limitations in our study. First, this is a cross-sectional study with a small sample size. The association of EDPs and vascular calcification needs further validation in future multicenter researches. Second, we cannot figure out the causal link between EDPs and AAC progression because of a lack of following up. Finally, the model was based on limited clinical information. EDPs are biologically active and can interact with various cells and participate in physiological and pathological processes [40], thus *in vitro* and *in vivo* studies are needed to elucidate its relationship with AAC in PD patients.

In conclusion, EDPs improved the risk stratification of AAC and severe AAC among PD patients and provided novel insights into EDPs on AAC as a potential indicator.

## Supplementary Material

Supplemental Material

## Data Availability

The data that support the findings of this study are openly available in figshare at http://doi.org/10.6084/m9.figshare.13172660.
