# Supplementary material for: Association between serum elastin-derived peptides and abdominal aortic calcification in peritoneal dialysis patients: a cross-sectional study
Source: Ren Fail. 2021 May 17;43(1):860–8. doi: 10.1080/0886022X.2021.1918163 (PMC8143601; doi:10.1080/0886022X.2021.1918163)
Supplement: Supplemental Material [file IRNF_A_1918163_SM2765.pdf]

Supplementary Methods

Association Between Serum Elastin-derived Peptides and Abdominal Aortic Calcification in Peritoneal Dialysis Patients：A Cross-sectional study

CT Protocol

Supine abdominal CT scans were obtained without intravenous contrast agents (Philips Brilliance 64 spiral CT machine). The tube voltage was 120 kVp, and the modulated tube current ranges from 20mA to 500mA. Images were reconstructed with a thickness of 1 mm and a matrix of 512 * 512.

Enzyme linked immunosorbent assay (ELISA)

The levels of serum EDPs of 126 PD patients and 30 healthy controls were determined with commercial ELISA kits (SK00806-01). Human soluble EDPs present in serum samples compete with a fixed amount of biotinylated human soluble elastin for sites on an antibody specific against EDPs. After the enzyme reaction, add stop solution to each well and the reaction yields a yellow product. The intensity of the color is inversely proportioned to the amount of EDPs in serum samples. Determine the OD value using a microplate reader set to 450nm, and establish a standard curve and read off the sample values.

Establishment of nomogram to visualize the risk model of AAC and severe AAC

Nomogram was generated by the "rms" R package (version 6.0-0) in R project environment (version 4.0.2, Foundation for Statistical Computing, Vienna, Austria) for the clinical use of

the regression model. To use the nomogram, find the position corresponding to the coordinate axis of each variable, draw a line upwards to the point axis for the number of points. Add all the points together to get a total point and find the position corresponding to the total point axis. Draw a line downwards to the risk axis to determine the risk of AAC (Fig.S2) and severe AAC (Fig.S3). In addition, we also validate the models using the bootstrap validation method.

After model fitting, we also validate the models by bootstrap validation and established a calibration curve. Please see the attached files named ‘AAC-calibration.pdf’ and ‘severe AAC-calibration.pdf’. The x-axis means the predicted risk (range 0 – 1), and the y-axis means the actual risk (range 0 - 1). To some extent, the models may underestimate or overestimate the risk of AAC or severe AAC.

Tables

Table S1. Comparisons of medications between PD patients with or without AAC

	PD		*P*
	Non-AAC<br>N=41	AAC<br>N=85	
RAS inhibitor n,(%)	33 (80.5)	67 (78.8)	1.000
β-receptor inhibitor n,(%)	12 (29.3)	26 (30.6)	1.000
αβ-receptor inhibitor	18 (43.9)	35 (41.2)	0.848
CCB n, (%)	39 (95.1)	71 (83.5)	0.088
diuretic n,(%)	10 (24.4)	15 (17.6)	0.475
Calcium phosphate binder	1 (2.4)	2 (2.4)	1.000
phosphate-lowering medications	22 (53.7)	42 (49.4)	0.706
vitamin D and its analogs	15 (36.6)	26 (30.6)	0.546
statin	13 (31.7)	15 (17.6)	0.108
UA- lowering treatments	2 (4.9)	8 (9.4)	0.497

AAC indicates abdominal aortic calcification; PD, peritoneal dialysis; RAS, renin-angiotensin-aldosterone system. CCB, calcium channel blocker; UA, uric acid.

Table S2. Comparisons of medications between PD patients with or without severer AAC

	PD		P
	Non severe AAC N=49	Severe AAC N=36	
RAS inhibitor n,(%)	36 (73.5)	31 (86.1)	0.188
β-receptor inhibitor n,(%)	15 (30.6)	11 (30.6)	1.000
αβ-receptor inhibitor	18 (36.7)	17 (47.2)	0.377
CCB n,(%)	41 (83.7)	30 (83.3)	1.000
diuretics n,(%)	12 (24.5)	3 (8.3)	0.083
Calcium phosphate binder	1 (2.0)	1 (2.8)	1.000
phosphate-lowering medications	22 (44.9)	20 (55.6)	0.384
vitamin D and its analogs	14 (28.6)	12 (33.3)	0.643
statin	5 (10.2)	10 (27.8)	0.046*
UA lowering treatments	5 (10.2)	3 (8.3)	1.000

AAC indicates abdominal aortic calcification; RAS, renin-angiotensin-aldosterone system; CCB, calcium channel blocker; UA, uric acid. *$p < 0.05$.

Table S3. Correlation analyses of EDPs with other parameters in PD patients.

	R	*P*
Age,year	0.511	< 0.001 ***
SBP, mmHg	0.141	0.230
DBP, mmHg	0.154	0.191
BMI, kg/m^2	0.002	0.985
PD vintage, month	0.178	0.159
Total Kt/V	0.208	0.099
HB, g/L	0.003	0.977
NE, 10^9/L	0.099	0.397
PLT, 10^6/L	0.003	0.983
TG, mmol/L	0.156	0.179
TC, mmol/L	0.236	0.040 *
HDL, mmol/L	0.147	0.204
LDL, mmol/L	-0.030	0.798
Cr, µmol/L	-0.023	0.847
β2-MG, mg/L	-0.014	0.918
UA, mmol/L	-0.078	0.504
Alb, g/L	-0.017	0.886
Ca, mmol/L	0.029	0.805
P, mmol/L	-0.053	0.648
hs-CRP, mg/L	0.167	0.149
ALP, U/L	0.382	0.001 **
iPTH, ng/L	-0.125	0.325
Annular, degree	0.488	< 0.001 ***

Spearman correlation analysis was used to find association of EDPs with other parameters.

R indicates the correlation coefficient. * $p < 0.05$, ** $p < 0.01$, *** $p < 0.001$.

Table S4. Univariate logistic regression analyses of variables with AAC in PD patients.

	Bcoff.	SE	Wald	OR	95% CI	P
Age,year	0.115	0.023	25.860	1.122	1.073	$< 0.001^{***}$
Male n,(%)	-0.21	0.385	0.296	0.811	0.381-1.726	0.587
SBP, mmHg	-0.008	0.011	0.555	0.992	0.971-1.013	0.456
DBP, mmHg	-0.043	0.017	6.414	0.958	0.927-0.990	0.011^{*}
BMI, kg/m^2	-0.059	0.060	0.960	0.943	0.839-1.060	0.943
T2DM n, (%)	0.405	0.696	0.340	2.500	0.384-5.864	0.560
CVD n, (%)	0.560	0.825	0.460	1.750	0.347-8.823	0.498
PD vintage, month	0.040	0.011	14.311	1.041	1.019-1.062	$< 0.001^{***}$
Total Kt/V	0.723	0.459	2.478	2.060	0.838-5.065	0.115
HB, g/L	-0.019	0.013	2.305	0.981	0.957-1.006	0.129
NE, 10^9/L	0.131	0.143	0.839	1.140	0.861-1.510	0.360
PLT, 10^6/L	-0.005	0.003	2.487	0.995	0.988-1.001	0.115
TG, mmol/L	-0.070	0.160	0.190	0.933	0.682-1.275	0.663
TC, mmol/L	-0.039	0.175	0.050	0.962	0.682-1.356	0.824
HDL, mmol/L	0.103	0.626	0.027	1.108	0.325-3.782	0.869
LDL, mmol/L	-0.101	0.245	0.170	0.904	0.559-1.1462	0.680
Cr, µmol/L	-0.001	0.001	1.546	0.999	0.998-1.000	0.214
β2-MG, mg/L	0.017	0.018	0.857	1.017	0.981-1.054	0.355
UA, mmol/L	-0.008	0.003	6.958	0.992	0.986-0.998	0.008^{**}
Alb, g/L	-0.077	0.044	3.039	0.926	0.8501.010	0.081
Ca, mmol/L	0.260	1.016	0.066	1.297	0.177-9.494	0.798
P, mmol/L	-0.771	0.484	2.541	0.463	0.179-1.194	0.111
hs-CRP, mg/L	-0.004	0.049	0.008	0.996	0.904-1.097	0.931
ALP, U/L	0.013	0.013	0.005	6.092	1.003-1.024	0.014^{*}
iPTH, ng/L	0.007	0.007	1.048	1.007	0994-1.021	0.306
EDPs, ng/mL	0.086	0.021	16.477	1.090	1.046-1.137	$< 0.001^{***}$

$^{*}p < 0.05$, $^{**}p < 0.01$, $^{***}p < 0.001$.

Table S5. Univariate logistic regression analyses of variables with severe AAC in patients with aortic calcification.

	B.coff	SE	Wald	OR	95% CI	P
Age,year	0.069	0.022	9.697	1.072	1.026-1.120	0.002 **
Male n,(%)	0.005	0.45	0.000	1.005	0.416-2.429	0.992
SBP, mmHg	0.021	0.014	2.339	1.021	0.994-1.049	0.126
DBP, mmHg	0.017	0.022	0.623	1.017	0.975-1.061	0.430
BMI, kg/m^2	0.058	0.081	0.515	1.060	0.904-1.244	0.473
T2DM n, (%)	0.095	0.710	0.018	1.100	0.274-4.423	0.893
CVD n, (%)	0.651	0.798	0.665	1.917	0.401-9.153	0.415
PD vintage, month	0.047	0.011	19.146	1.048	1.026-1.070	< 0.001 ***
Total Kt/V	-1.029	0.553	3.469	0.357	0.121-1.055	0.063
HB, g/L	-0.001	0.015	0.001	0.999	0.971-1.029	0.973
NE, 10^9/L	0.049	0.153	0.103	1.050	0.778-1.419	0.749
PLT, 10^6/L	0.001	0.004	0.049	1.001	0.993-1.009	0.825
TG, mmol/L	-0.246	0.229	1.158	0.782	0.500-1.224	0.282
TC, mmol/L	-0.005	0.200	0.001	0.996	0.672-1.473	0.980
HDL, mmol/L	0.508	0.714	0.508	1.663	0.411-6.734	0.476
LDL, mmol/L	-0.075	0.272	0.076	0.928	0.545-1.581	0.783
Cr, µmol/L	0.002	0.001	5.405	1.002	1.000-1.004	0.010 *
β2-MG, mg/L	0.071	0.024	8.750	1.074	1.024-1.126	0.003 **
UA, mmol/L	0.005	0.004	2.262	1.005	0.998-1.012	0.133
Alb, g/L	-0.064	0.044	2.067	0.938	0.861-1.023	0.151
Ca, mmol/L	0.796	1.071	0.552	2.216	0.272-18.063	0.457
P, mmol/L	1.348	0.649	4.315	3.851	1.079-13.740	0.038 *
hs-CRP, mg/L	0.036	0.061	0.350	1.037	0.920-1.169	0.554
ALP, U/L	0.000	0.005	0.001	1.000	0.991-1.01	0.970
iPTH, ng/L	0.017	0.008	5.546	1.017	1.001-1.033	0.033 *
EDPs, ng/mL	0.045	0.019	5.397	1.046	1.007-1.086	0.020 *

* $p < 0.05$, ** $p < 0.01$, *** $p < 0.001$.

Figures

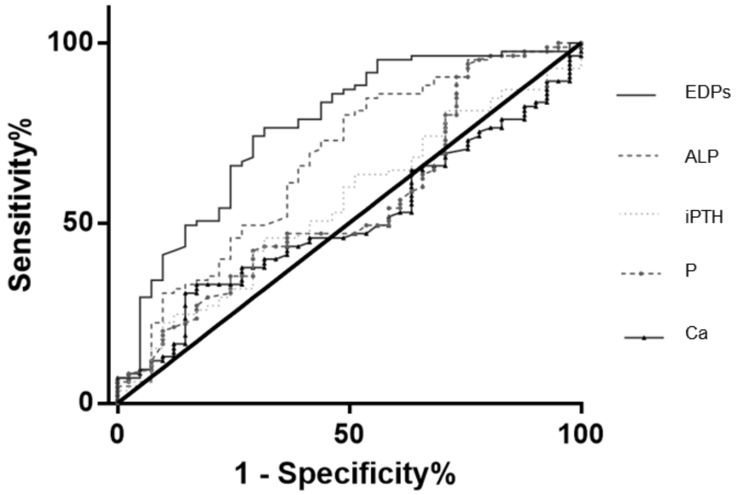

Fig.S1 Receiver operating characteristic curves of EDPs and mineral metabolism parameters for AAC. The prediction value of EDPs was the largest among these parameters, with an AUC of 0.767. The respective AUC of mineral parameters including Ca, P, ALP, and iPTH was 0.504, 0.554, 0.672, and 0.552. EDPs indicate elastin-derived peptides; P, phosphate; ALP, alkaline phosphatase; Ca, calcium; iPTH, intact parathyroid hormone.

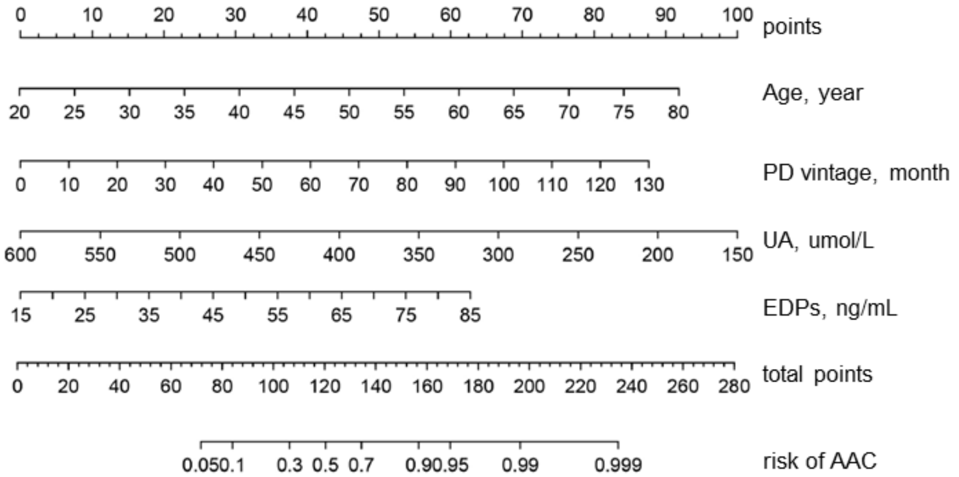

Fig.S2 A nomogram of risk factors to predict AAC in PD patients. AAC indicates abdominal aortic calcification; EDPs, elastin-derived peptides; and UA, uric acid.

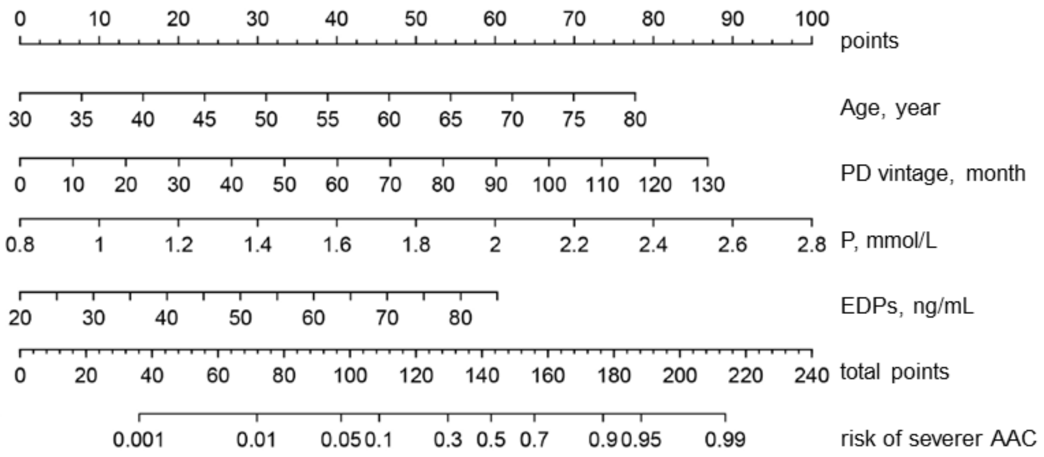

Fig.S3 A nomogram of risk factors to predict severe AAC in PD patients. AAC indicates abdominal aortic calcification; EDPs, elastin-derived peptides; PD vintage, peritoneal dialysis duration; and P, phosphate.
